# Model based on GA and DNN for prediction of mRNA-Smad7 expression regulated by miRNAs in breast cancer

**DOI:** 10.1186/s12976-018-0095-8

**Published:** 2018-12-29

**Authors:** Edgar Manzanarez-Ozuna, Dora-Luz Flores, Everardo Gutiérrez-López, David Cervantes, Patricia Juárez

**Affiliations:** 1 Universidad Autónoma de Baja California, Carretera Transpeninsular Ensenada-Tijuana 3917 Colonia Playitas, C.P. 22860 Ensenada, B.C. Mexico; 2 Centro de Investigación Científica y de Educación Superior de Ensenada, Carretera Ensenada-Tijuana No. 3918, Zona Playitas, C.P. 22860 Ensenada, B.C. Mexico

**Keywords:** Deep Neural Networks, Genetic Algorithms, miRNA, regulation, mRNA, Smad7

## Abstract

**Background:**

The Smad7 protein is negative regulator of the TGF-β signaling pathway, which is upregulated in patients with breast cancer. miRNAs regulate proteins expressions by arresting or degrading the mRNAs. The purpose of this work is to identify a miRNAs profile that regulates the expression of the mRNA coding for Smad7 in breast cancer using the data from patients with breast cancer obtained from the Cancer Genome Atlas Project.

**Methods:**

We develop an automatic search method based on genetic algorithms to find a predictive model based on deep neural networks (DNN) which fit the set of biological data and apply the Olden algorithm to identify the relative importance of each miRNAs.

**Results:**

A computational model of non-linear regression is shown, based on deep neural networks that predict the regulation given by the miRNA target transcripts mRNA coding for Smad7 protein in patients with breast cancer, with R^2^ of 0.99 is shown and MSE of 0.00001. In addition, the model is validated with the results *in vivo* and *in vitro* experiments reported in the literature. The set of miRNAs hsa-mir-146a, hsa-mir-93, hsa-mir-375, hsa-mir-205, hsa-mir-15a, hsa-mir-21, hsa-mir-20a, hsa-mir-503, hsa-mir-29c, hsa-mir-497, hsa-mir-107, hsa-mir-125a, hsa-mir-200c, hsa-mir-212, hsa-mir-429, hsa-mir-34a, hsa-let-7c, hsa-mir-92b, hsa-mir-33a, hsa-mir-15b, hsa-mir-224, hsa-mir-185 and hsa-mir-10b integrate a profile that critically regulates the expression of the mRNA coding for Smad7 in breast cancer.

**Conclusions:**

We developed a genetic algorithm to select best features as DNN inputs (miRNAs). The genetic algorithm also builds the best DNN architecture by optimizing the parameters. Although the confirmation of the results by laboratory experiments has not occurred, the results allow suggesting that miRNAs profile could be used as biomarkers or targets in targeted therapies.

**Electronic supplementary material:**

The online version of this article (10.1186/s12976-018-0095-8) contains supplementary material, which is available to authorized users.

## Background

The development of cancer is given by the loss of regulation in cellular processes such as growth, death, proliferation, differentiation, adhesion, migration among others in many types of cells due to the accumulation of mutations or drastic changes in deoxyribonucleic acid (DNA) [1]. The transforming growth factor beta 1 (TGF-β1) is overexpressed in breast cancer [2] and regulates these processes through the initiation of the TGF-β cellular signaling pathway, which induces the genetic expression and the cellular processes as response to stimuli on the outside of the cell [3, 4].

The TGF-β signaling pathway initiate when an activated TGF-β1 ligand binds to a receptor II (TβRII). The latter in turn recruits and activates receptor I (TβRI) to form the receptor complex dependent on the activated ligand. The activated TβRI phosphorylates the Smads proteins regulated by the receptor (R-Smads). The cooperating Smads (Co-Smads) bind to activated R-Smads to integrate the SMAD complex that translocate to the nucleus. Once inside the nucleus, it binds with DNA binding proteins and act as transcription factors that regulate the expression of target genes [4].

The Smad7 protein is part of the inhibitory Smads (I-Smads) which are antagonistic proteins and interrupt the transduction process of the TGF-β signaling pathway by proteasomal degradation of the receptor complex dependent on the activated ligand, preventing phosphorylation of the R-Smads, avoiding the formation of functional SMAD complexes and blocking the binding of the SMAD complex to DNA [4].

The above establishes a negative regulation loop between the transforming growth factor beta (TGFβ1) as a promoter and Smad7 as an inhibitor of the TGF-β signaling pathway.

On the other hand, ribonucleic acid microRNAs (miRNAs) are small non-coding RNAs with a length between 21 and 25 nucleotides involved in the regulation of cell division, development, oncogenesis, apoptosis, among other processes by repressing the protein translation and degradation of the messenger ribonucleic acid (mRNA) transcripts. The miRNAs are transcribed by RNA polymerase II as part of polyadenylated and protected primary transcripts (pri-miRNA), which can be of coding or non-coding protein. The primary transcript is cleaved by the enzyme Drosha ribonuclease III to produce a stem loop precursor miRNA of approximately 70-nt (pre-miRNA), which is further cleaved by ribonuclease dicer cytoplasmic to generate the mature miRNA and the antisense miRNA star (miRNA*). The mature miRNA is incorporated into an RNA-induced silencing complex (RISC), which recognizes target mRNAs through imperfect or perfect base pairing with the miRNA [5].

There is reported evidence on the relationship of the miRNAs and the inhibitory proteins of the TGF-β signaling pathway, where the overexpression of miR-21 or the low expression of Smad7 promotes the fibroblast formation associated with carcinoma [6]. Overexpression of miR-21 can inhibit the proliferation of rat renal tubular epithelial cells [5]. miR-21 promotes the proliferation and invasion of breast cancer cells by suppressing Smad7 [7]. Besides, Yan et al. [8] observed that nine miRNAs were more than twofold up-regulated versus seven miRNAs under expressed in tumors compared with the adjacent normal tissue. While Apostolos et al. demonstrated that the expression of miR-21, miR-210 and miR-221 has a significant role in the development of primary triple negative breast cancer [9].

All the aforementioned, suggests the existence of regulation of the expression of the Smad7 protein given by the miRNAs on the mRNA coding of the Smad7 protein (mRNA-Smad7) and the possible impact on the negative regulation loop of signaling pathway of TGF-β mediated by TGFβ1 and Smad7. For this process, a non-linear dynamic is observed, since there are multiple interactions between miRNAs and mRNAs, thus it is feasible to approach it from the complexity point of view.

Since, computational models have been used previously to predict protein expression regulation given by miRNAs. In [10] a computational approach based upon emerging biomedical and biological ontologies and semantic technologies to investigate the important roles of microRNA, mRNA regulation on glucocorticoid resistance in pediatric acute lymphoblastic leukemia.

Machine learning based models to predict potential disease-related long noncoding RNAs (lncRNAs) has been developed based on Laplacian Regularized Least Squares [11], semantic similarity [12, 13], and Naive Bayesian classifier [14].

Moreover, biological network-based and random walk with restart as predictor models has been developed based on lncRNA-lncRNA functional similarity [15], lncRNAs and PCGs expression profiles in prostate cancer and protein interaction datasets [16], integrate three networks miRNA-associated lncRNA-lncRNA crosstalk network, disease-disease similarity network, and known lncRNA-disease association network [17], using three networks, disease-disease similarity network, lincRNA-lincRNA similarity network and known lincRNA-disease association network [18], integrate known lncRNA-disease associations, lncRNA expression profiles, lncRNA functional similarity, disease semantic similarity [19], coding-non-coding gene-disease bipartite network based on known disease genes and lncRNA-disease associations and further implemented a propagation algorithm on this bipartite network to infer the underlying lncRNA-disease associations [20].

In addition, computational framework based on disease genes to predict lncRNA-disease association based on the known disease-related genes/miRNAs and the relationships between lncRNAs and genes/miRNAs has been developed. In [21] computational framework infers that there could be potential associations between this lncRNAs with diseases related with these human tissues. Furthermore, it could obtain related diseases for non-tissue-specific lncRNAs based on disease–gene associations and gene-lncRNA co-expression relationship. Ten lncRNAs predicted to be associated with vascular smooth muscle cells were selected for further experimental validation to test the accuracy of the method. As a result, eight of ten lncRNAs (80%) were confirmed [22]. Chen [23] developed a novel inference computational model based on HyperGeometric distribution for LncRNA-Disease Association inference (HGLDA) by integrating miRNA-disease associations and lncRNA-miRNA interactions. Furthermore, constructed a model of lncRNA functional similarity calculation based on the information of miRNA (LFSCM) to calculate lncRNA functional similarity combining disease semantic similarity, miRNA-disease associations and lncRNA-miRNA interactions.

Deep neural networks (DNN) based on a metaphoric form in the human nervous system, are information processing systems composed of simple elements highly interconnected and have been used successfully for prediction in systems of non-linear dynamics [24]. These have been used to identify miRNAs associated with breast cancer phenotypes [25], diagnosis of tumors and candidate identification for therapy based on gene expression [26], precursor classification of microRNA [27], identification of profiles of expression in stage II tumors associated with aggressive disease [28], also to identify biologically relevant miRNAs associated with specific breast cancer phenotypes and expression of miRNAs in rectal cancer as predictors of response to neoadjuvant chemoradiation therapy [29].

DNN design intrinsically implies the challenge of determining its architecture (number of hidden layers, number of nodes per layer, output layer, etc.), establishing the input data set, defining the validation method in the training process, among other characteristics, that integrate the DNN with the best predictive capacity. This challenge is addressed as an automatic search problem over a solution space.

On the other hand, genetic algorithms (GA) are an automatic optimization technique based on the principles of evolution postulated by Darwin which establish natural selection and the adaptation of individuals to the environment as evolutionary elements along with convergence toward the best solutions in a search space. GAs, in conjunction with DNNs, have been used in predicting renal colic in emergency settings [30], in the optimization of weights in the DNN training process [31], among others.

Based on the above, the aim of this research is to develop a computational model based on DNN and GA to predict the regulation given by the miRNA target mRNA-Smad7 in patients with breast cancer. Particularly, GA is used for feature selection and optimizing the parameters of DNN architecture.

## Methods

### Data collection and processing

Based on the objective of this study, we identified 179 miRNAs that interact with the mRNA-Smad7 database in the mirDB [20, 32, 33], microRNA [34], and MiRTarBase [35]. As well, a set of 1074 samples expression files of patients with breast cancer was downloaded from the project website “The Cancer Genome Atlas” [36], each set of files in a sample contains the base and normalized expression of the miRNAs, the mRNAs, among other data that were not considered for this work. The initial dataset was integrated by the base values of mRNA-Smad7 and the base values of the miRNAs of all the files set [37] with size of 1074 records (rows) × 180 fields (columns) (a field with the expression of mRNA-Smad7 and 179 expressions of miRNAs) values.

### Data preprocessing

Given the initial dataset in each sample, the existence of mRNA-Smad7 expression and the existence of miRNAs expression were validated, from which it was obtained that the 1074 samples have an expression of mRNA-Smad7 and that a total of 39 miRNAs do not have expression in the samples, reducing from 179 to 140 miRNAs (Additional file 1), therefore the size of the dataset was reduced to 1074 records by 141 fields. Starting from this new dataset, the expression of each miRNA was validated in all the samples and 41 miRNAs were removed, as they do not contribute statistically in the explanation of the variability of mRNA-Smad7 expression, given that its expression values in 75% samples (third quartile) is lower than two or has a mode equal to zero and the frequency of the mode greater than 15% samples (Additional file 2), the size of the dataset changed to 1074 records by 100 fields (Additional file 3).

Once the dataset was defined, it was normalized and used in linear regression models including the generalized linear model, regression trees, assembly of regression trees, support vector machines, and Gaussian processes regression. The best overall performance was from Gaussian process regression with R2 = 0.12 and MSE = 0.014077. Thus, no function was found that adjusted the dataset (Additional file 4).

The expression vectors of each miRNA were considered as time series of data and were transformed by the discrete Meyer wavelet [38] to eliminate possible noise in the data, defined in Eq. (1). A characteristic of the wavelet transform is to preserve the original signal after the transformation, this transformation shows better performance in relation with conventional filtering methods applied on genomic data [39], they have been used for filtering biological data signals [40], in the classification of tumors using microarrays of gene expression data [41], among others.

The simple linear regression technique was applied to the dataset to identify and eliminate the exact linear relationship and / or the high correlation between the miRNAs and thus validate the assumption of multicollinearity, obtaining as a result the non-existence of collinearity between the miRNAs (results omitted in this document).

The miRNAs expression was scaled in the range from − 1 to 1 (Eq. 2) and thus tied with the function of sigmoid activation of the DNN. The expression of the mRNA-Smad7 was scaled in the range from 0 to 1, since the activation function is linear, and the expected values are in that range.

1 $$ {\displaystyle \begin{array}{c}\psi \left(\omega \right)=\left\{\begin{array}{c}\frac{1}{\sqrt{2\pi }}\sin \left(\frac{\pi }{2}v\left(\frac{3\left|\omega \right|}{2\pi }-1\right)\right){e}^{\frac{j\omega}{2}}\kern0.75em if\kern0.5em \frac{2\pi }{3}<\left|\omega \right|<\frac{4\pi }{3},\\ {}\frac{1}{\sqrt{2\pi }}\cos \left(\frac{\pi }{2}v\left(\frac{3\left|\omega \right|}{4\pi }-1\right)\right)\ {e}^{\frac{j\omega}{2}}\kern0.5em if\kern0.5em \frac{4\pi }{3}<\left|\omega \right|<\frac{8\pi }{3},\\ {}0\kern10.5em otherwise\kern8em \end{array}\right.\\ {} where:\kern1.5em v(x)=\left\{\begin{array}{c}0\kern0.75em if\kern0.5em x<0,\kern1.75em \\ {}x\kern0.75em if\kern0.5em 0<x<1,\\ {}1\kern0.75em if\kern0.5em x>1\kern2em \end{array}\right.\end{array}} $$

**Fig. 1:**
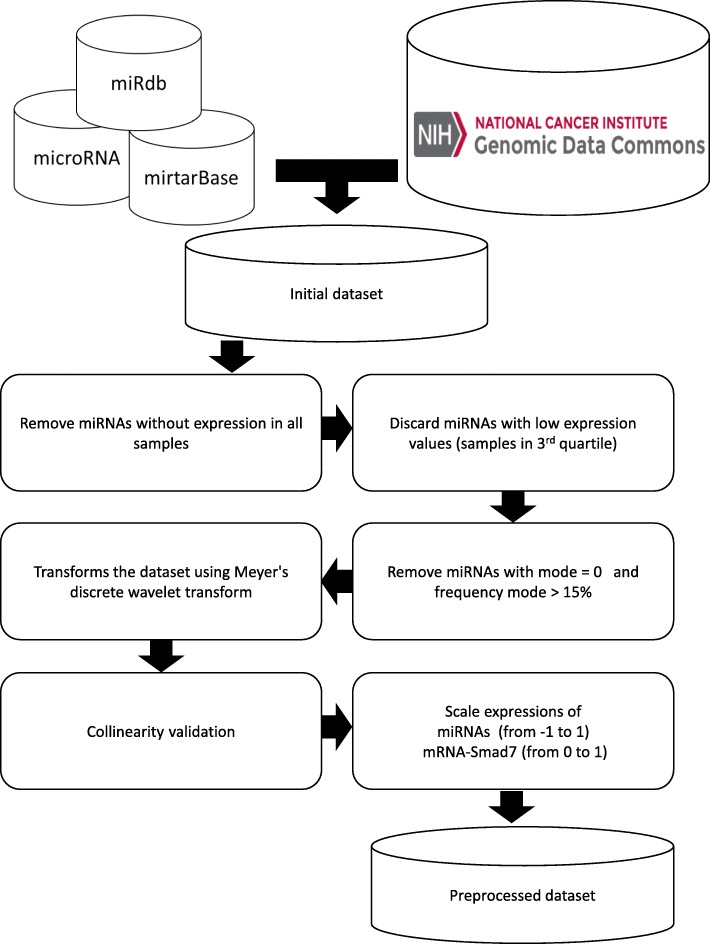
Steps involved in the data collection and preprocessing

Finally, the preprocessed dataset was integrated with 1074 samples that have mRNA-Smad7 expression as a dependent variable and the expression of 99 miRNAs as independent predictor variables (See Fig. 1).

2 $$ f(x)=\frac{x-\mu (x)}{\max (x)-\mu (x)} $$

## Proposed model

### Method of evolutionary search

After developing different linear regression models, including generalized linear model, regression trees, assembly of regression trees, support vector machines, and Gaussian processes regression, R^2^ values were from 0.70 to 0.92; however, according to some authors [26, 27], those values could be increased using DNNs.

**Fig. 2:**
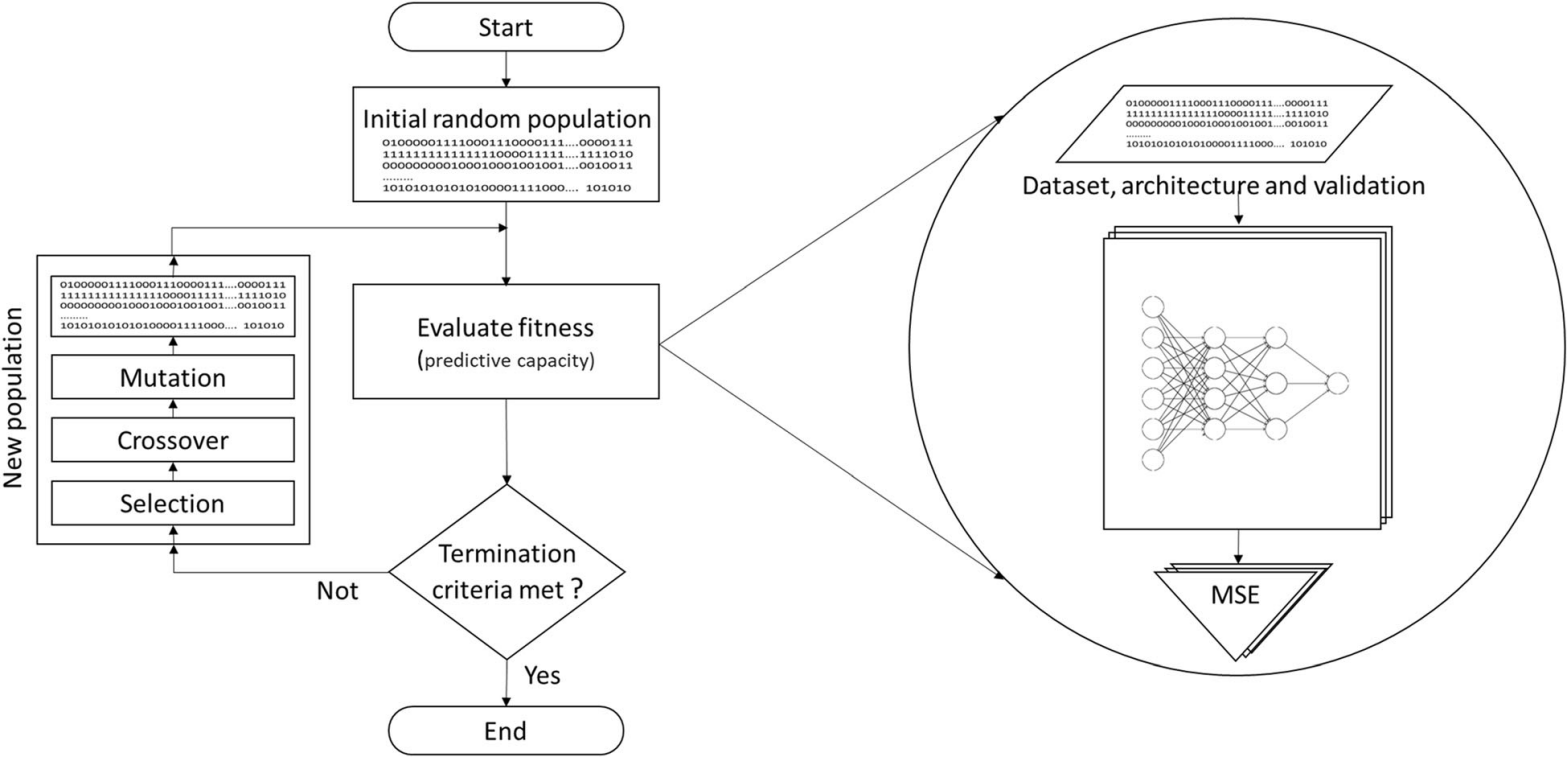
Evolutionary method of the data set, architecture and validation (EMDAV)

The design of the predictive model was treated as an optimization problem of automatic search over a solution space, where each point (solution) represents a predictive model with its own characteristics and predictive capacity. To tackle that problem, an evolutionary method based on GA was developed as an automatic search optimization technique to automatically design evolutionary DNNs as predictive models, as shown in Fig. 2.

This method establishes the mechanisms to evolve the input dataset, the DNN architecture and the validation method for the DNN training process, for this work, it is called Evolutionary Method of the Dataset, Architecture, and Validation (EMDAV). The EMDAV was executed 20 times, the maximum evolution was up to 100 generations, with a stopping criterion of five generations without significant change in the predictive capacity of the evaluated models.

### Genetic algorithm

The search for a model with better predictive capabilities was treated as a minimization problem (see Algorithm 1) with the mean squared error (MSE) as an adaptation value, using a binary type genetic algorithm with an initial population of 50 individuals with a uniform crossing operator with 80% of probability, linear range selection operator, uniform random mutation operator with 10% probability and elitism factor of 10%. In order to find a better adjustment to allow the evolution of desired characteristics by a process of parameters tuning, experiments were carried out with the selection factors with four levels (Linear rank, nonlinear rank, roulette wheel and tournament) and crossing with two levels (Single point and uniform), the treatment was the evolution of GA.

The selection operator in linear rank and the uniform crossover operator were established given that they are the levels of the treatment factors with higher R^2^ and lower MSE. Similarly, the population size of 50 individuals was established through the evolution of GA with population size of 20, 50 and 100 individuals. The base coding for the individuals of the population is of variable length, composed of 99 bits that encode the set of input data where a value of one (1) means that the miRNA is part of this set, while a value of zero (0) that it is not; one bit for the validation methods (0 for hold-out and 1 for k-folds), the maximum number of hidden layers encoded in six bits and the maximum number of nodes for each hidden layer encoded in ten bits, as described in Eq. (3).

3 $$ individual\ length=99 bits+1 bit+6 bits\ast 10 bits $$

The number of hidden layers results from the conversion to decimal of 6 bits + 1 decimal avoiding the existence of 0 layers, in the same way the number of nodes per hidden layer is given by the conversion to decimal of 10 bits + 1 decimal. The variability of the length of the individual is given by the last two terms of Eq. (3), which establish the necessary length for the maximum values in both terms, in other cases, starting from bit 107 only the first (*n* + 1) * 10 bits are processed, where *n* + 1 is the number of hidden layers and therefore 10 * (64 - (*n* + 1)) bits are not part of the individual in question.

### Deep neural networks

Each GA individual was transformed into a DNN with architecture given by the input data set of variable size of miRNAs, sigmoid transfer function (Eq. 4) between input layers and hidden layers, linear transfer function between the last hidden layer and the output layer and were trained with the mini-batch gradient descent optimization algorithm called RMSprop [42] with a fixed batch size of 50 and with a fixed learning rate of 0.01.

A set of 20 runs were executed with hold-out validation and 10 times with k-folds validation up to a maximum of 5000 epochs for both scenarios. For each run, the predictive capacity, determined by the MSE, was calculated by randomly divided the dataset into three subsets, training (50%), validation (25%), and testing (25%).

To avoid overfitting, a stop threshold of 10 gradient updates was set in the validation process. As well as, a 50% discard rate of nodes and their incoming and outgoing connections between the last hidden layer and the output layer as dropout value.

4 $$ S(t)=\frac{L}{1+{e}^t} $$

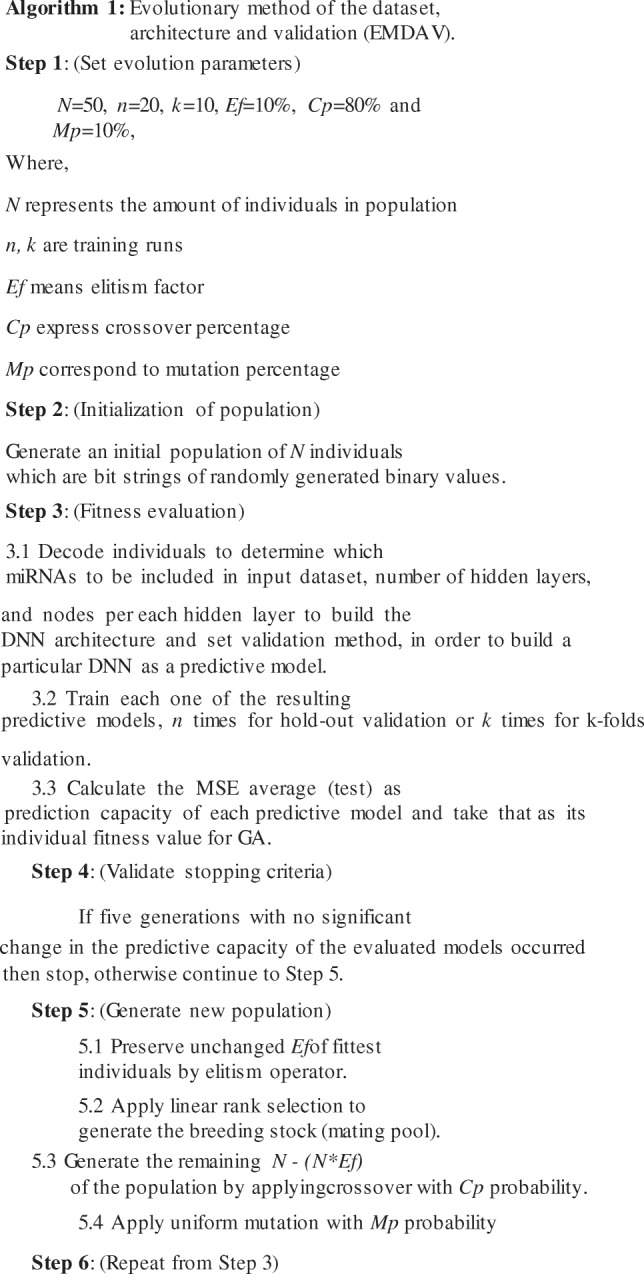

## Results and discussion

In this section we discuss the results obtained by the EMDAV method and the prediction model based on deep neural networks.

### Evolution of the EMDAV method

In the different executions of the EMDAV method, a maximum of 24 generations were obtained, in the execution with the greatest number of generations 17,760 evaluations of phenotypes were made up to the generation 24 + 5, reaching the stopping criteria, of which 1560 used k-folds validation with 10 folds and the other remaining 16,200 with validation hold-out and 20 repetitions. The variability of the validation method feature was segregated from generation 14, the architecture was established in generation 21, and the remaining generations were used exclusively for the evolution of the input data set.

Table 1 shows the ten models with greater predictive capacity and it can be seen that in the evolution of the characteristics that there is a tendency to converge towards the same values.

**Table 1:** Best predictive model based on DNN

Generation	Individual	miRNAs count	Validation	Hidden layers	MSE	R^2^
23	17	45	Hold-out	42:63	0.003877	0.9275
19	16	53	Hold-out	42:63	0.004425	0.9224
21	28	53	Hold-out	42:63	0.003982	0.9174
21	6	52	Hold-out	42:63	0.003612	0.9167
17	36	52	Hold-out	42:63	0.003668	0.9139
18	20	54	Hold-out	42:63	0.004021	0.9138
22	7	50	Hold-out	42:63	0.004431	0.9137
20	8	55	Hold-out	46:61	0.004826	0.9128
17	5	53	Hold-out	42:63	0.004176	0.9119
19	25	55	Hold-out	42:63	0.004045	0.9112

The results shown in Table 1 only represent a small part of the experiments performed in the algorithm set-up process. This process had to be carried out carefully due to the computational cost, leading for example to the execution of exploratory executions on incremental search spaces in the range of 4 hidden layers and 64 nodes per layer up to 64 hidden layers and 1024 nodes per layer with both types of validation and a single fold or repetition. For all explored evolutions, the GA converged to the first architecture shown in Table 1. Given this, the final exploration was performed with 20 repetitions for hold-out and 10 folds for k-fold and the results were consistent. Which allows establishing the predictive model based on DNN described in the next section.

### Predictive model based on DNN

**Fig. 3:**
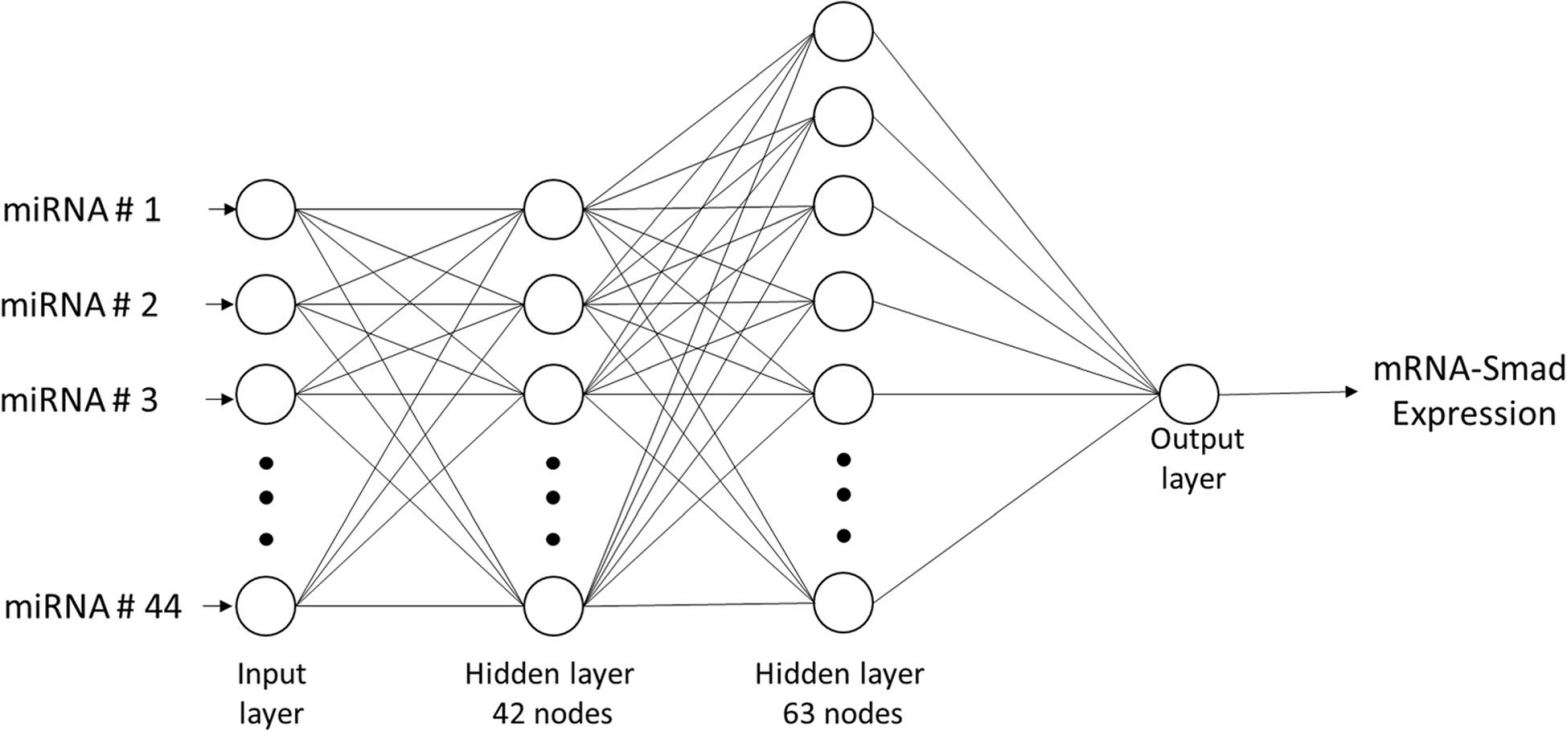
Reduced graphical representation of DNN predictive model

Figure 3 shows the predictive model based on DNN with architecture of two hidden layers with 42 and 63 nodes respectively, type of hold-out validation, an input data set composed of 44 miRNAs as shown in Table 2 and it such as multi-layer perceptron (MLP), with logistic activation function (Eq. 5) and trained with the resilient backpropagation with weight backtracking algorithm called Rprop+ [43], predicts the regulation given by the miRNAs target the mRNA transcripts coding for the Smad7 protein in patients with breast cancer with R^2^ = 0.99 and MSE = 0.00001.

**Table 2:** Predictive model evolved dataset

hsa-let-7c	hsa-let-7d	hsa-let-7 g	hsa-mir-100	hsa-mir-107	hsa-mir-10b
hsa-mir-126	hsa-mir-140	hsa-mir-145	hsa-mir-146a	hsa-mir-148a	hsa-mir-149
hsa-mir-15b	hsa-mir-183	hsa-mir-185	hsa-mir-191	hsa-mir-200c	hsa-mir-205
hsa-mir-21	hsa-mir-212	hsa-mir-22	hsa-mir-224	hsa-mir-23a	hsa-mir-26b
hsa-mir-29a	hsa-mir-29c	hsa-mir-30a	hsa-mir-32	hsa-mir-33a	hsa-mir-34a
hsa-mir-375	hsa-mir-378	hsa-mir-425	hsa-mir-429	hsa-mir-497	hsa-mir-503
hsa-mir-92b	hsa-mir-93	hsa-mir-125a	hsa-mir-15a	hsa-mir-20a	hsa-mir-27a
hsa-mir-374a	hsa-mir-625

### Relative importance of miRNAs to regulate mRNAS-Smad7 expression

The relative importance of each miRNAs on the expression of mRNA-Smad7 was evaluated using the Olden algorithm [44, 45], which is based on the weights of the connections of each node in the DNN and considers the magnitude and the direction, where the weight represents the intensity and the direction the excitation of the signal, therefore, a greater weight with positive direction, represents a greater relative importance and increases the predictive capacity of the model.

The results shown in Table 3 correspond to the average values of the miRNAs that increase the predictive capacity of the model since they are of great intensity and positive direction. Obtained from 20 runs of the predictive model such as MLP, with logistic activation function and trained with Rprop+.

**Table 3:** Relative importance positive on mRNAS-Smad7 from miRNAs

miRNAs	% Relative	miRNAs	% Relative	miRNAs	% Relative
hsa.mir.146a	12.00%	hsa.mir.29c	4.13%	hsa.let.7c	1.52%
hsa.mir.93	10.71%	hsa.mir.497	3.95%	hsa.mir.92b	1.32%
hsa.mir.375	10.18%	hsa.mir.107	3.66%	hsa.mir.33a	1.18%
hsa.mir.205	7.93%	hsa.mir.125a	3.40%	hsa.mir.15b	1.10%
hsa.mir.15a	7.75%	hsa.mir.200c	3.16%	hsa.mir.224	1.09%
hsa.mir.21	7.72%	hsa.mir.212	1.97%	hsa.mir.185	0.74%
hsa.mir.20a	7.70%	hsa.mir.429	1.68%	hsa.mir.10b	0.22%
hsa.mir.503	5.31%	hsa.mir.34a	1.59%		

Considering the information contained in the samples of patients with breast cancer taken from the TGCA project, the results show that the miRNAs hsa-mir-146a, hsa-mir-93, hsa-mir-375, hsa-mir-205, hsa-mir-15a, hsa-mir-21, hsa-mir-20a, hsa-mir-503, hsa-mir-29c, hsa-mir-497, hsa-mir-107, hsa-mir-125a, hsa-mir-200c, hsa-mir-212, hsa-mir-429, hsa-mir-34a, hsa-let-7c, hsa-mir-92b, hsa-mir-33a, hsa-mir-15b, hsa-mir-224, hsa-mir-185 and hsa-mir-10b integrate a profile that critically regulates the expression of mRNA-Smad7 and Smad7 protein in breast cancer and could be used as biomarkers or as targets in targeted therapies.

5 $$ f(x)=\frac{L}{1+{e}^{-k\left(x-x0\right)}} $$

These results are consistent with some previously reported studies that associate miRNAs with mRNA-Smad7 and Smad7 protein in breast cancer, such as the hsa-mir-146a that is an oncomiR that regulates the expression of mRNA and the Smad7 protein in non-alcoholic fibrous steatohepatitis [46] and it is over expressed in the plasma of patients with breast cancer [47]. Hsa-mir-93 is an oncomiR part of cluster 106b-25 involved in the mesenchymal epithelial transition suppressing the expression of Smad7 and activating the TGF-Beta signaling pathway in breast cancer [48]. Hsa-mir-21 is an oncomiR that promotes breast cancer proliferation and migration through the suppression of Smad7, which improves the epidermal growth factor signaling pathways (EGF) and TGF-Beta [7]. The cluster miR424–503 contains the oncomiR hsa-mir-503, which is expressed in metastatic breast cancer and suppresses the expression levels of Smad7 and Smurf2 [49]. Hsa-mir-497 is an oncomiR low expressed in breast cancer [9], the mRNA-Smad7 is a target of this and are negatively correlated in breast cancer [50].

Similarly, some studies that associate miRNAs with the mRNA-Smad7 and the Smad7 protein in different types of cancer or other malignancies are reported, such as: hsa-mir-375 is an oncomiR associated with single-polymorphisms nucleotide of Smad7 in colorectal cancer (CRC) and collectively can be considered as non-invasive biomarkers in the detection and diagnosis of CRC [51]. Hsa-mir-15a is an oncomiR with no reports associated with breast cancer and in infections of the hepatitis B virus, it regulates apoptosis and tumorigenesis based on the regulation of Smad7 [52]. Hsa-mir-212 activates hepatic stellate cells and promotes fibrosis in the liver by suppressing Smad7 [53]. Hsa-mir-92b promotes the progression of hepatocellular carcinoma via the repression of Smad7 [54]. Hsa-mir-15b targets mRNA-Smad7 in angiogenesis in myocardial infarction [55]. Hsa-mir-185-3p predicts the radiosensitivity of nasopharyngeal carcinoma and modulates the growth and apoptosis of cancer cells by regulating Smad7 [56].

On the other hand, some miRNAs are reported associated only with breast cancer, such as hsa-mir-205 is a tumor suppressor in breast cancer inhibits cell proliferation and anchorage independent growth as well as cell invasion, ErbB3 and vascular endothelial growth factor A (VEGF-A) are direct targets [57], significantly underexpressed in breast cancer [9]. Hsa-mir-20a were significantly overexpressed in breast cancer [58]. Hsa-mir-29c is an oncomiR underexpressed in breast cancer [8]. Hsa-mir-107 is an oncomiR underexpressed in stem cells of breast cancer [59], associated with strong probabilities of recurrence and with overall reduced OS values for triple-negative breast cancer [60] the overexpression accelerates the tumor progression of HCC in vitro and in vivo through its new target gene CPEB3 [61]. Hsa-mir-125a is a tumor suppressor, were significantly downregulated in her2-positive breast cancers, overexpression in an erb2-dependent cancer cell line (skbr3) suppressed her2 and her3 transcript and protein levels, which decreased cell motility and invasiveness [62]. Hsa-mir-200c is underexpressed in breast cancer [63].

The miRNAs hsa-mir-429, hsa-mir-34a, hsa-let-7c and hsa-mir-33a are not reported in the literature as directly associated with Smad7. In protein expression levels, interruption or suppression processes of the gene expression at transcription and translation levels are implicated, as well as proteosomal degradation or protein proteolysis, among other processes.

In particular, TGF-beta signaling pathway, the suppression of gene expression at the transcriptional level can be given by co-repressors (c-Ski / SnoN, Evi1, among others).

However, elucidating the relationship of all the mechanisms involved in the expression of the Smad7 protein is beyond the scope of this research.

As described, in the manuscript [6–9], there is evidence of the relationship between the miRNAs and the inhibitory proteins of the TGF-β signaling pathway. The results presented in Table 3, represent the relative importance for each miRNAs for the predictive model with the presented dataset.

Finally, the results obtained by the predictive model have been consistent with previously published works. This lays the foundations for the hypotheses development that confirming the results with laboratory experiments. Similarly, it establishes a precedent for the application of this methodology to alternative datasets that provide evidence to support the generalization of the results. However, these research hypotheses are part of the set of perspectives and future work in the research line.

## Conclusions

We develop a non-linear regression model based on DNN using Gas to predict the expression of mRNA-Smad7 regulated by the miRNAs, validated through the results of experiments in vivo and in vitro reported in the literature. Such hybrid system is capable of finding both features and the architecture of the best neural network, including number of layers, the neurons per layer, validation method in the training process, and training algorithm. In summary, GAs has been used in the proposed model of deep neural networks for two main tasks: determining features inputs and designing the structure of the deep neural network.

An evolutionary search method based on binary GA called EMDAV is presented, where each individual is manifested in DNN and the input data set, architecture, and training validation are evolved as characteristics that define the predictive capacity of the model. This method is able to find a prediction model based on DNN that fits the biological data with R^2^ = 0.99 and MSE of 0.00001.

A profile of critical regulation is established for the expression of mRNA-Smad7 and the expression of the Smad7 protein in breast cancer integrated by hsa-mir-146a, hsa-mir-93, hsa-mir-375, hsa-mir-205, hsa-mir-15a, hsa-mir-21, hsa-mir-20a, hsa-mir-503, hsa-mir-29c, hsa-mir-497, hsa-mir-107, hsa-mir-125a, hsa-mir-200c, hsa-mir-212, hsa-mir-429, hsa-mir-34a, hsa-let-7c, hsa-mir-92b, hsa-mir-33a, hsa-mir-15b, hsa-mir-224, hsa-mir-185 and hsa-mir-10b.

The relative importance granted to every miRNAs has been supported by previously reported studies [8, 9, 47–63]. However, the miRNAs set associated with mRNA-Smad7 in breast cancer is integrated by five miRNAs (hsa-mir-146a, hsa-mir-93, hsa-mir-21 hsa-mir-503 and hsa-mir-497) and they accumulate 40% of the relative importance assigned by the predictive model.

On the other hand, it has the set integrated by hsa-mir-375, hsa-mir-15a, hsa-mir-212, hsa-mir-92b, hsa-mir-15b and hsa-mir-185-3p, are reported as associated with mRNA-Smad7 in other types of cancer or malignancies and their cumulative relative importance of 23%.

Both sets are disjoint and contain the miRNAs directly related to mRNA-Smad7, in conjunction they accumulate 63% of the relative importance granted by the predictive model.

From the above, it is possible to establish hypotheses and explore, on the one hand, the possible relationship between the miRNAs selected by the predictive model, but not previously reported, in association with mRNA-Smad7 in breast cancer. On the other hand, the impact on other cancers of the interaction between mRNA-Smad7 and miRNAs reported in association with breast cancer and selected by the predictive model.

In the same sense, for the set of miRNAs associated with breast cancer but not associated with the mRNA-Smad7, experiments need to be carried out to elucidate the possible relationship with mRNA-Smad7.

Future work includes the application of this methodology to other data sets related to breast cancer or other diseases, as well as the development of laboratory experiments to confirm the relevance of each miRNA in the regulation of mRNA-Smad7.

## Additional files

Additional file 1: Initial dataset. A table with 1074 records (samples of patients with breast cancer) by 141 fields (mRNA-Smad7 expression and expression values of 140 miRNAs). (XLS 2480 kb)

Additional file 2: Dataset descriptive statistics. Dataset with descriptive statistic as a tool to discard miRNAs based on expression levels. (XLS 55 kb)

Additional file 3: Preprocessed dataset. The dataset after preprocessing, integrated by 1074 samples including mRNA-Smad7 expression as the dependent variable and the expression of 99 miRNAs as independent predictor variable. (XLS 1880 kb)

Additional file 4: Regression models not function adjusted to dataset. Regression models results. The best overall performance is from squared exponential Gaussian process regression with R^2^ = 0.12 and MSE = 0.014077. (XLS 30 kb)
